# A Case of Premature Delivery, Induced by Dysentery, without the Usual Pain of Labor

**Published:** 1871-06

**Authors:** C. D. Smith

**Affiliations:** Newnan, Ga.


*A Case of Premature Delivery, Induced by Dysentery, Without the usual Pain of Labor.* By C. D. Smith, of Newnan, Ga.

---

I was called upon by Mr. G——, of this county, on the 8th of June, to prescribe for his wife, who, he informed me, was suffering with an attack of dysentery. I learned from him the characteristic discharges from the bowels were very frequent, attended with severe tormina and tenesmus; that she was in the ninth month of pregnancy, and expected confinement in about two weeks. My opinion as to the result of the dysentery upon the uterus, in this case, was that delivery would be premature, and in the end proved to be correct. I prescribed for her small doses of calomel in combination with Dover's powder, directing a powder to be given at intervals of three to four hours, and persevered in until the secretions changed their appearance. The medicine had the effect desired, and was followed with Castor Oil and Tincture Opii, which produced one or two good fœcal operations, mixed with bilious matter, greatly to the relief of my patient; but the amelioration of symptoms proved to be only temporary. After an interval of ten or twelve hours, her husband came in haste to consult me again as to her condition. He stated that she was suffering greatly with griping pains in her bowels, the bloody mucus discharges having returned with nausea of stomach and complete prostration of strength. With a view to the relief of pain and procuring rest, I gave him four powders of Sulphate Morphia, containing one-fourth grain each, with directions to give one every hour until she was relieved. After the second dose had been given it had the happy effect so much desired. She became quiet, fell into a gentle sleep, and rested some hours without any interruption. On visiting her in the morning, I found her with but little fever, and the dysenteric symptoms almost entirely relieved. She expressed herself as feeling a great deal better; and indeed, so great a change had really taken place with her, that we were almost ready to conclude that our predictions would not be verified. After the expiration of six or eight hours, however, we were summoned again in great haste. After arriving and entering the room, we found our patient now evidently in the first stage of labor, uterine pains and contractions coming on at regular intervals. On making a vaginal examination, we found the os tiusæ slightly dilated, the presentation natural, the external parts soft and flacid; everything favorable for a safe delivery. I was of the opinion, from all the indications, that it would not probably be long before her delivery. Strange to say, I had barely been seated before being called again in haste to the bed-side. Making a second examination, I found the head of the child pressing the perineum, and by one simple expulsive effort, without any expression of pain, the complete delivery of the child was effected.

This is the second case of the kind occurring in a practice of more than twenty years. Nature, inexorable in her laws, has doomed poor woman to suffer the throes and agonies of child-birth. But our patient seems to have been an exception to some extent, and more fortunate than thousands of her sex. She informs us that in giving birth to eight children she has always had a quick and easy time. It was certainly so with her in this instance. No untoward or unpleasant symptoms have occurred since her confinement. Mother and child are both doing well.

